# Growth arrest and DNA damage-inducible proteins (GADD45) in psoriasis

**DOI:** 10.1038/s41598-021-93780-x

**Published:** 2021-07-16

**Authors:** Pedro Rodríguez-Jiménez, Lola Fernández-Messina, María C. Ovejero-Benito, Pablo Chicharro, Paula Vera-Tomé, Alicia Vara, Danay Cibrian, Pedro Martínez-Fleta, María Jiménez-Fernández, Inés Sánchez-García, Mar Llamas-Velasco, Francisco Abad-Santos, Francisco Sánchez-Madrid, Esteban Dauden, Hortensia de la Fuente

**Affiliations:** 1 Department of Dermatology, Instituto de Investigación Sanitaria del Hospital Universitario de la Princesa (IIS-IP), Universidad Autónoma de Madrid (UAM), Madrid, Spain; 2 Department of Immunology, Instituto de Investigación Sanitaria del Hospital Universitario de la Princesa (IIS-IP), Universidad Autónoma de Madrid (UAM), 28006 Madrid, Spain; 3 CIBER de Enfermedades Cardiovasculares (CIBERCV), Madrid, Spain; 4 Clinical Pharmacology Department, Instituto de Investigación Sanitaria del Hospital Universitario de la Princesa (IIS-IP), Universidad Autónoma de Madrid (UAM), Madrid, Spain; 5 Teófilo Hernando, Instituto de Investigación Sanitaria del Hospital Universitario de la Princesa (IIS-IP), Universidad Autónoma de Madrid (UAM), Madrid, Spain; 6 Departamento de Ciencias Farmacéuticas y de la Salud, Facultad de Farmacia, Universidad San Pablo-CEU, (CEU Universities), Madrid, Spain; 7 Centro Nacional de Investigaciones Cardiovasculares, CNIC, Instituto de Salud Carlos III, Madrid, Spain; 8 Centro de Investigación Biomédica en Red de Enfermedades Hepáticas y Digestivas (CIBERehd), Instituto de Salud Carlos III, Madrid, Spain

**Keywords:** Cell biology, Immunology

## Abstract

The interplay between T cells, dendritic cells and keratinocytes is crucial for the development and maintenance of inflammation in psoriasis. GADD45 proteins mediate DNA repair in different cells including keratinocytes. In the immune system, GADD45a and GADD45b regulate the function and activation of both T lymphocytes and dendritic cells and GADD45a links DNA repair and epigenetic regulation through its demethylase activity. Here, we analyzed the expression of GADD45a and GADD45b in the skin, dendritic cells and circulating T cells in a cohort of psoriasis patients and their regulation by inflammatory signals. Thirty patients (17 male/13 female) with plaque psoriasis and 15 controls subjects (7 male/8 female), were enrolled. Psoriasis patients exhibited a lower expression of GADD45a at the epidermis but a higher expression in dermal infiltrating T cells in lesional skin. The expression of GADD45a and GADD45b was also higher in peripheral T cells from psoriasis patients, although no differences were observed in p38 activation. The expression and methylation state of the GADD45a target UCHL1 were evaluated, revealing a hypermethylation of its promoter in lesional skin compared to controls. Furthermore, reduced levels of GADD45a correlated with a lower expression UCHL1 in lesional skin. We propose that the demethylase function of GADD45a may account for its pleiotropic effects, and the complex and heterogeneous pattern of expression observed in psoriatic disease.

## Introduction

Growth Arrest and DNA Damage-inducible proteins, namely GADD45a, GADD45b and GADD45g are small proteins of 18–20 kDa molecular weight. These proteins exert their functions by protein–protein interactions both in the nucleus and the cytoplasm, and are involved in DNA repair, cell proliferation, survival and differentiation among other biological processes^1^. GADD45 family members are associated with stress responses through the stimulation of p38-JNK mitogen-activated protein kinases (MAPK) that mediate important functions in both innate and adaptive immune cells. In T cells, T cell receptor (TCR) engagement induces GADD45b and GADD45g expression, promoting a sustained p38 activation and subsequently, leading to IFN-γ production and Th1 differentiation^2,3^. In T cells, GADD45b is also induced in response to the pro-inflammatory cytokines IL-12 and IL-18^4^. In contrast to GADD45b and GADD45g, GADD45a is expressed in resting T cells. GADD45a has been shown to inhibit the alternative p38 activation pathway, since T cells from GADD45a-deficient displayed constitutive p38 activation^5^. Regarding the innate immune system, dendritic cells (DCs) from GADD45a-deficient mice exhibited a reduced production of Th1-related cytokines, IL-12 and a lower expression of co-stimulatory molecules, such as CD40^6^. Moreover, GADD45b−/− DCs showed reduced IL-12 and IL-6 cytokine production, and GADD45b-deficient mice exhibited an impaired Th1 response^3^. With these effects on the immune system, it is not surprising that GADD45 proteins have been associated with autoimmune-related diseases. Indeed, GADD45a-deficient mice spontaneously develop an autoimmune disease characterized by the presence of autoantibodies against double-stranded (ds) DNA^5^. Moreover, GADD45b-deficient mice showed exacerbated experimental autoimmune encephalomyelitis, while GADD45b and GADD45g double-deficient mice develop a spontaneous lymphoproliferative disease^7^.

On the other hand, GADD45 expression has been associated with DNA repair in keratinocytes^8^. GADD45 protein expression induced by UV light in human keratinocytes has been linked to the production of reactive oxygen species (ROS) and the activation of NADPH oxidase, likely as a mechanism to counter-regulate the oxidative damage^9,10^. In this sense, GADD45a is implicated in the G2/M cell cycle checkpoint in UV-irradiated cells, and GADD45a-deficient transgenic mice show genomic instability^11^. Interestingly, GADD45a acts as a link between DNA repair and epigenetic gene regulation by mediating demethylation^12^. GADD45a controls the expression of the matrix metallopeptidase (MMP)-9 in keratinocytes, through the recruitment of thymine-DNA glycolase and the induction of demethylation of the MMP9 promoter^13^. In addition, silencing of GADD45a in endothelial cells reduce the expression of the ubiquitin carboxyl-terminal hydrolase L1 (UCHL1) via UCHL1 promoter methylation^14^. Interestingly, decreased UCHL1 expression has been detected in skin lesions with itch of psoriasis patients, while an increased number of DCs expressing UCHL1 has been described in atopic dermatitis patients^15^.

We hypothesized that GADD45 molecules could play a role in the development of psoriasis, a disease where the interaction of keratinocytes and immune cells is pivotal, and where epigenetic modifications cause aberrant increases in epidermal thickness, keratinocyte differentiation, proliferation and inflammation^16^.

## Material and methods

### Human patients and samples collection

This study was approved by the Institutional Review Board (IRB)/Independent Ethics Committee of Hospital de la Princesa, according to the Declaration of Helsinki Principles. After giving informed consent, 15 control individuals and 30 patients with untreated plaque psoriasis were enrolled. Clinical and demographic characteristics are shown in Table 1. Patients were eligible for the study if they were adult candidates to systemic therapy. The following washout periods were established: 14 days for topical corticosteroids, 28 days for systemic treatment including corticosteroids, methotrexate, cyclosporine, acitretin or phototherapy and 3 months for biologic agents. From each psoriasis patient, two non-sun-exposed cutaneous biopsies (10 mm) were taken, one from lesional psoriatic skin and another one from apparently healthy skin (non-lesional skin). At the same time, 20 ml of peripheral venous blood were extracted. Normal leftover skin samples and peripheral venous blood samples were obtained from 15 surgical patients. Each biopsy was cut in half; one piece was snap frozen for RNA isolation, and the other one included in OCT and stored at −80 °C until processing for immunofluorescence staining.

**Table 1:** Clinical characteristics of psoriasis patients and healthy subjects.

	Psoriasis patients	Healthy subjects
Subjects (n)	30	15
Sex (M/F)	17/13	7/8
Age (years)	56.4 (25–89)	54.9 (33–90)
PASI	14.98 (7.8–31.2)	

Results are expressed as mean (min–max). M, male; F, female; PASI, psoriasis area and severity index.

### Quantitative RT-PCR

GADD45a, GADD45b, UCHL1 and IFN-γ mRNA expression levels were determined by quantitative reverse transcription polymerase chain reaction (RT-PCR). Total RNA was isolated from skin samples, peripheral blood CD4^+^ T cells and monocyte-derived (mo) DCs, using the TRIzol reagent (Invitrogen), and following the manufacturer's instructions. Briefly, one microgram of RNA was transcribed to cDNA and amplified with the specific primers pairs using GoTaq qPCR Master Mix (Promega, WI USA). Real‐time (RT)–PCR was performed in a CFX384 Real‐time System (Bio‐Rad) using SYBR Green PCR Master Mix (Applied Biosystems, Carlsbad CA). The data were analyzed using StepOne Plus Software (Applied Biosystems). GADD45a, GADD45b, UCHL1 and IFN-γ mRNA levels were normalized to GAPDH levels and expressed as relative levels.

### Immunofluorescence staining

Skin OCT sections of 5 μm were fixed (formaldehyde 4%), permeabilized (Triton X-100 0.2%) and blocked with 100 µg/ml of human γ-globulin (Sigma-Aldrich, St Louis MO, USA) and a 1:100 dilution of donkey serum (Sigma-Aldrich) in phosphate buffer solution (PBS). Skin sections were then incubated over-night with 5 μg/ml goat anti-human GADD45a (Abcam)and mouse anti-human CD3 antibodies (Dako), followed by donkey anti-goat (DAG) Alexa Fluor 488 and DAM Alexa Fluor 555. Finally, cell nuclei were counterstained with DAPI. Negative controls were performed with omission of the primary antibody. Sections were examined with a Leica DMR immunofluorescence microscope under the same acquisition conditions. Images were analyzed using the ImageJ software (http://imagej.softonic.com) and GADD45a levels were determined on regions of interest (ROIs), drawn for CD3^+^ lymphocytes.

### Peripheral blood T cells and monocyte derived DCs (moDCs) isolation and culture

Peripheral blood mononuclear cells (PBMCs) were obtained by density gradient in Ficoll Hypaque and CD4^+^ T cells were isolated by negative selection using magnetic microbeads (Miltenyi Biotec Bergisch Gladbach, Germany). Where indicated, CD4^+^ T cells were incubated for 24 h in the presence of IL-12 (10 ng/ml) plus IL-18 (10 ng/ml). For moDCs, PBMCs were allowed to adhere for 30 min at 37°C, and plastic adhered cells were cultured for 5 days in complete RPMI medium supplemented with 500 U/ml GM-CSF (Peprotech) and 10 ng/ml IL-4 (R&D systems). On day 6, 10 ng/ml of LPS were added and cells were harvested 24 h after incubation for analysis.

### Expression of p38 (pTryr180/pThr183) in CD4^+^ T cells analysis by flow cytometry

Isolated CD4^+^ T cells were incubated with anti-CD3 and anti-CD28 antibodies (10 µg/ml and 5 µg/ml respectively) for 30 min at 4ºC. Thereafter, anti-mouse Fc was added and incubated for an additional 30 min at 4°C and then immediately incubated at 37°C. After 15 min, cells were fixed, permeabilized and stained with mouse anti-human p38 (Becton–Dickinson®), following manufacturer’s instructions and analyzed in a FACScanto flow cytometer (BD Bioscience).

### GADD45a expression by flow cytometry

PBMCs (1 × 10^6^ cells/ml) were incubated with Immunocult human CD3/CD28 T cell activator (Stemcell Technologies). For flow cytometry staining, cells were incubated with FcR human blocking reagent for 15 min at 4ºC and then incubated with Ghost Red 780 viability dye (TONBO, Biosciences), following manufacturer’s instructions. Cells were then incubated with PE-conjugated anti-CD11c, and APC-conjugated anti-CD3 antibodies (both from BD). Last, cells were incubated with FoxP3 Fix/Perm buffer and FoxP3 Perm buffer (Biolegend) following manufacturer’s instructions and incubated with anti-GADD45a Alexa fluor 488 (Santa Cruz Biotechnology) for 30 min at 4 °C. Cells were analyzed in a FACScanto Flow Cytometer. Expression of GADD45a was evaluated as mean fluorescence intensity on live, CD11c^_^ CD3^+^ cells. Gating strategy is shown in Supplementary Fig. 1.

### Western blot

PBMCs were seeded on a plastic surface to let monocytes adhere. After 30 min of incubation cells were recovered and monocyte-depleted PBLs (2 × 10^6^ cells) were incubated at 1 × 10^6^ cell/ml either in the presence or absence of human recombinant IL-12 (10 ng/ml) plus IL-18 (10 ng/ml) for 24 h. Total proteins were extracted using RIPA buffer (1%NP40, 0.5% sodium deoxycholate, 0.1% SDS in TBS) supplemented with protease and phosphatase inhibitor cocktail (Roche Diagnostics). Proteins were resolved by SDS-PAGE and transferred onto a PVDF membrane (Merck Millipore). After transfer, membranes were blocked with 5% non-fat dry milk and then incubated with rabbit anti-human GADD45b (Abcam) and mouse anti-human GADPH (Biologend) antibodies, followed by incubation with the appropriate horseradish peroxidase-conjugated secondary antibodies. Chemiluminescence detection was perfomed with Immobilon Crescendo Western HRP substrate (Millipore). Full length blots are provided in Supplementary Fig. 2. Images were analyzed using the ImageJ software (http://imagej.softonic.com).

### Methylation-specific PCR

DNA was isolated from skin samples using High Pure PCR template (Roche). Bisulfite conversion was performed with EZ DNA Methylation kit (Zymo Research) following the manufacturer’s instructions using 350 ng of DNA. After conversion, DNA was eluted in 12 μl. For positive controls and standard curve of methylated primers Bisulfite Converted Universal Methylated human DNA Standard (Zymo Research) was used. Standard curve of unmethylated primers was performed using DNA from a control subject. As control of conversion reaction, we used Universal Methylated human DNA Standard (Zymo Research). Primers used for methylation specific PCR were next: UCHL1 Forward 5′-TCG TAT TTA TTT GGT CGC GATC-3′, Reverse 5′-CTA TAA AAC GCC GAC CAA ACG-3′, unmethylated UCHL1 forward 5′-GGT TTG TAT TTA TTT GGT TGT GAT T-3′, Reverse 5′-CAA CTA TAA AAC ACC AAC CAA ACA-3′^17^. PCR was performed in 10 μl final volume using 25 ng of converted DNA, 0.3 mM each primer and 5 μl of GoTaq qPCR Master mix (Promega). To correct for DNA input, we used a primer pair specific for a sequence of beta-actin where no CpG sites are present and described previously. ActB Forward 5′-TGGTGATGGAGGAGGTTTAGTAAGT-3′, ActB Reverse 5’-AACCAATAAAACCTACTCCTCCCTTAA-3′^18^. Data were represented as methylation ratio:

$$ {\text{Methylation}}\;{\text{ratio}} = {{\frac{{{\text{UCHL1}}\;{\text{methylated}}}}{{{\text{Beta-actin}}}}} \mathord{\left/ {\vphantom {{\frac{{{\text{UCHL1}}\;{\text{methylated}}}}{{{\text{Beta-actin}}}}} {~\frac{{{\text{UCHL1}}\;{\text{unmethylated}}}}{{{\text{Beta-actin}}}}}}} \right. \kern-\nulldelimiterspace} {~\frac{{{\text{UCHL1}}\;{\text{unmethylated}}}}{{{\text{Beta-actin}}}}}} $$

### Methylation datasets

Datasets from DNA methylation arrays were obtained from the repository Gene Expression Omnibus (https://www.ncbi.nlm.nih.gov/geo/). We searched for available methylation datasets performed in psoriasis patients. Three datasets of psoriasis skin samples fulfilled these criteria: GSE63315^19^, GSE73894^20^ and GSE115797^21^. In these datasets, skin punch biopsies of 4 mm diameter were collected. Methylation was analyzed with an Illumina Infinium Human Methylation 450 k BeadChip array following manufacturing protocol.

Although the three selected studies fulfil the inclusion criteria, and were performed with the same sampling and technology, we would like to point out the heterogeneity regarding the objectives pursued in the three studies, and their differences: (1) in GSE63315 dataset 1, data come from 12 pre-UV irradiation moderate-to-severe psoriasis patients and 12 healthy controls; (2) GSE115797 dataset 3 contained lesional (L) and non-lesional (NL) samples from 24 moderate-to-severe plaque psoriasis patients; (3) GSE73894 dataset 2 combines both types of data including 114 samples of lesional skin, 41 of non-lesional skin and 62 of healthy subjects^19–23^.

### Statistical analyses

Data were analyzed with GraphPad Prism 5.0 (GraphPad Software, San Diego, CA, USA). Kruskal–Wallis and Mann–Whitney U-tests were used, as appropriate. Where indicated, Wilcoxon signed rank test was used to analyze paired data. The Spearman test was used for correlation analysis. Significance was set at *p < 0.05, **p < 0.01, ***p < 0.001.

Differentially methylated CpG sites between psoriasis and controls were detected by GEO2R analysis tool (https://www.ncbi.nlm.nih.gov/geo/geo2r/) a web-based program that employs the Bioconductor packages GEOQuery^24^ and limma^22^ in R, with the Benjamini–Hochberg false-discovery rate (FDR). Log_2_Fold-change of methylation was calculated in psoriasis lesional skin referred to control (psoriasis non-lesional skin or healthy controls’ skin). Thus, positive values indicate that the UCHL1 promoter is hypermethylated in psoriatic skin with respect to controls, while negative values indicate hypomethylation. Although we have analyzed epigenetic differences in all the methylation sites included in this array (485,000), we have focused on those sites located on CpG islands of the UCHL1 promoter that present a FDR adjusted *p* value lower than 0.05. We have selected these regions since CpG island hypermethylation is commonly associated with gene repression.

## Results

### GADD45 proteins exhibit a complex pattern of expression in psoriatic skin

**Figure 1:**
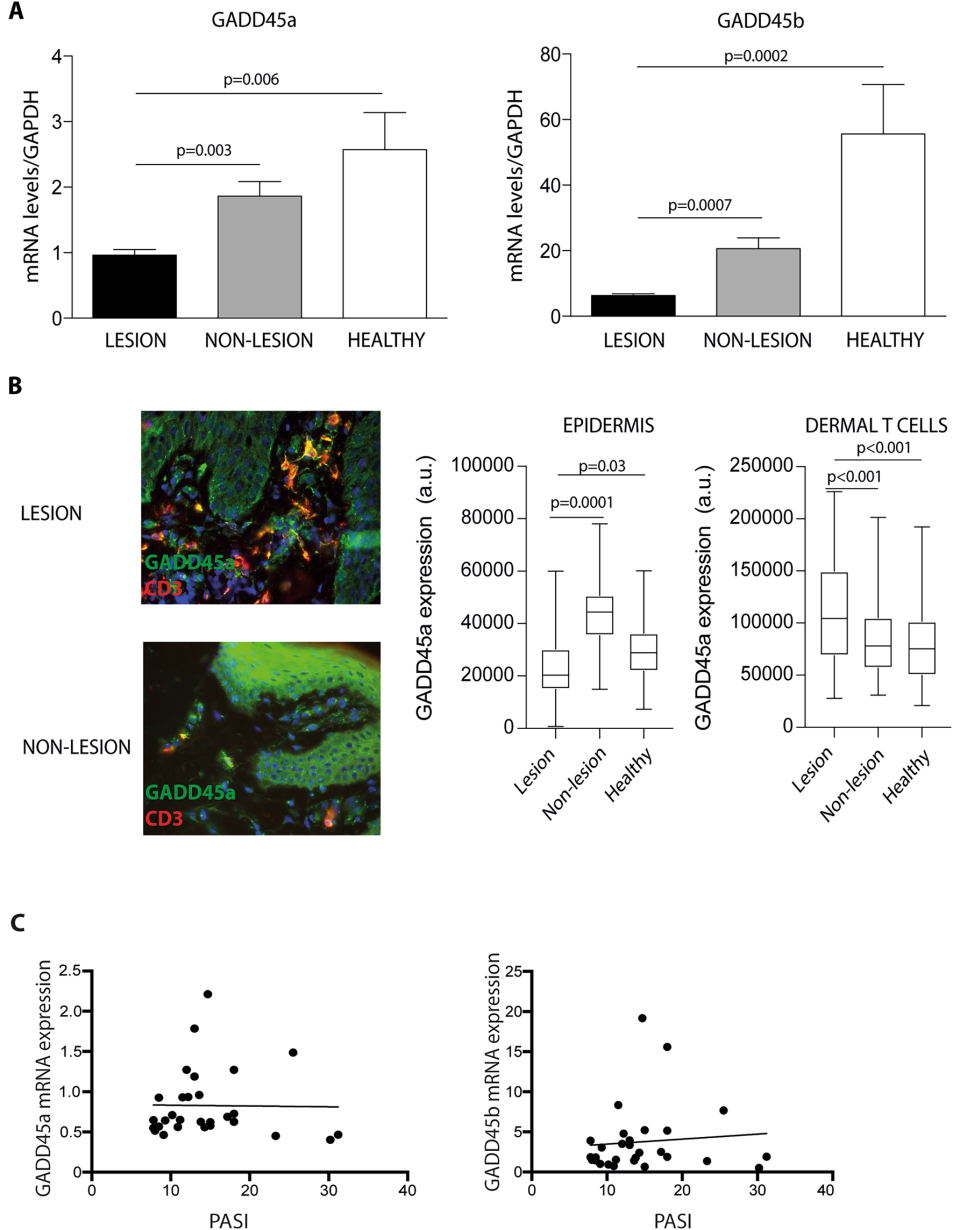
Lesional skin from psoriasis patients expresses low levels of GADD45a and GADD45b. (**A**) mRNA levels of GADD45a (left) and GADD45b (right) were analyzed by qRT-PCR in skin samples from 30 patients with psoriasis and 15 controls. GAPDH was used to normalize gene expression. Data were analyzed by one-way ANOVA followed by Tukey’s multiple comparisons test**.** (**B**) Representative staining of GADD45a (green) and CD3 (red) in skin samples from lesional skin and non-lesional skin. Quantification of immunofluorescence staining, fluorescence intensity of GADD45 in epidermal (left) and dermal (right) infiltrating T cells was calculated using the Image J software. Differences between groups were determined by one-way ANOVA followed by Tukey’s multiple comparisons test. (**C**) Correlation analysis of GADD45a and GADD45b mRNA expression in lesional skin of psoriasis patients and disease severity evaluated (PASI score).

To explore the role GADD45a and GADD45b in psoriasis, we first analyzed the expression of GADD45a and GADD45b genes in lesional and non-lesional skin samples from 30 patients (17 male/13 female) with plaque psoriasis and 15 control subjects (7 male/8 female). Patients enrolled had an average of Psoriasis Area and Severity Index (PASI) of 14.98, ranging from 7.8 to 31 (Table 1). Compared with non-lesional skin or healthy controls, lesional skin from psoriatic patients expressed lower levels of both GADD45a and GADD45b mRNA (Fig. 1A). Immunofluorescence assays showed the presence of GADD45a in keratinocytes and dermal T lymphocytes as indicated by the co-staining with CD3 (Fig. 1B). We observed a complex pattern of expression of GADD45a in the skin samples. While the levels of this protein in keratinocytes was diminished in psoriasis, its expression in dermal infiltrating lymphocytes was increased (Fig. 1B). GADD45a and GADD45b mRNA levels in lesional skin were not associated with disease activity (PASI), as shown by the correlation analysis (Fig. 1C).

**Figure 2:**
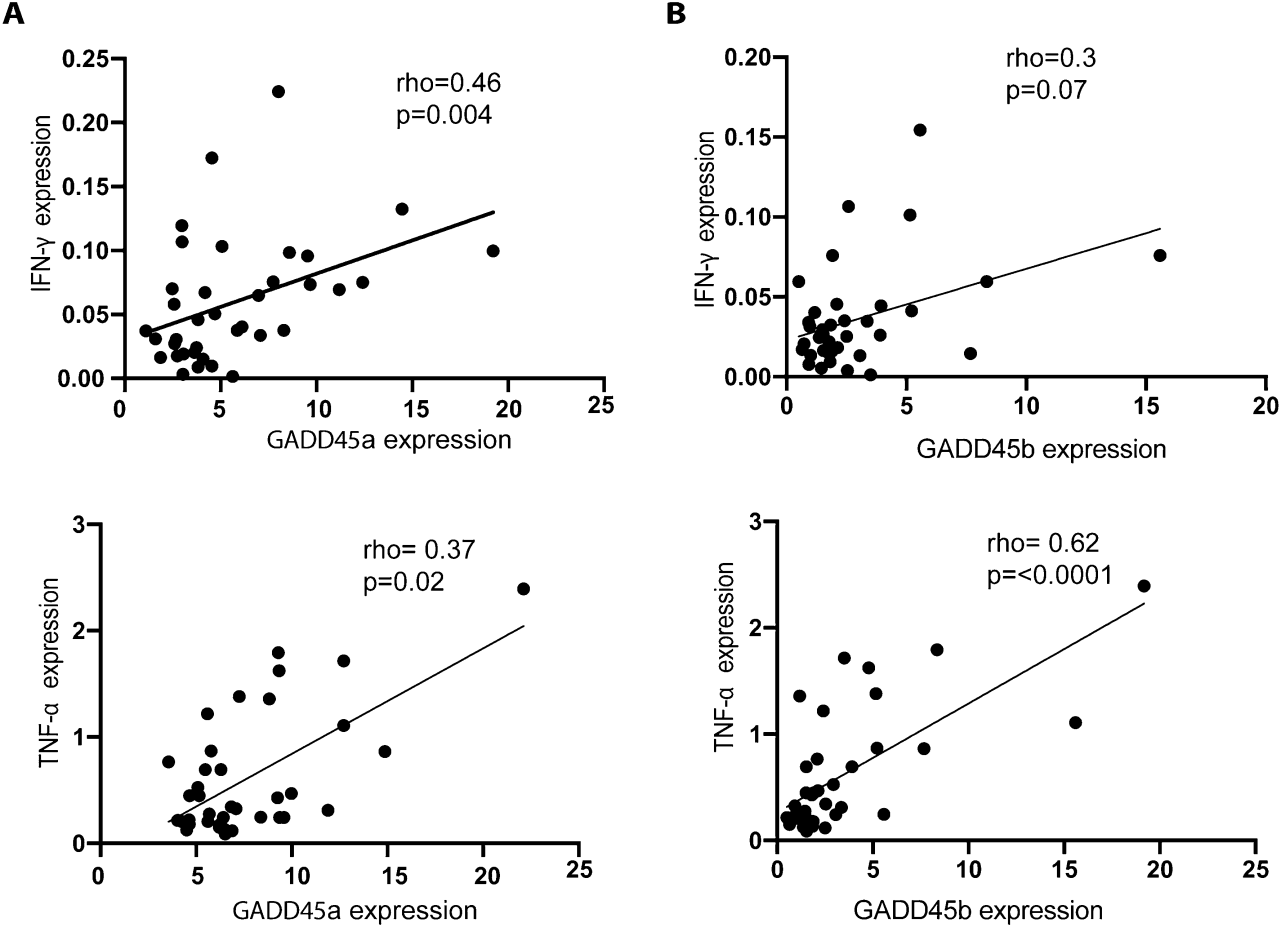
Expression of GADD45a correlates with IFN-γ and TNF-α in lesional skin of psoriasis patients. (**A**) Scatter graphs showing the correlation between mRNA levels of GADD45a and GADD45b and IFN-γ in lesional skin of psoriasis patients. (**B**) Scatter graphs showing the correlation between mRNA levels of GADD45a and GADD45b with TNF-α as in A. GADD45 expression was analyzed using qRT-PCR, normalized to GAPDH expression. Data were analyzed using Spearman´s test, n = 30.

As stated above, several inflammatory stimuli have been associated with the induction of GADD45 molecules. Thus, we postulated the existence of a possible association of GADD45 expression with the levels of the pro-inflammatory mediators IFN-γ or TNF-α. Our data showed a clear positive correlation between GADD45a and IFN-γ or TNF-α expression, both quantified by qRT-PCR (Fig. 2A). A positive correlation was also observed between GADD45b and TNF-α, however, the association between the levels of GADD45b and IFN-γ was not significant (Fig. 2B).

### Psoriasis patients circulating CD4^+^T cells express increased levels of GADD45 proteins, while immature moDCs exhibit low levels of these proteins

**Figure 3:**
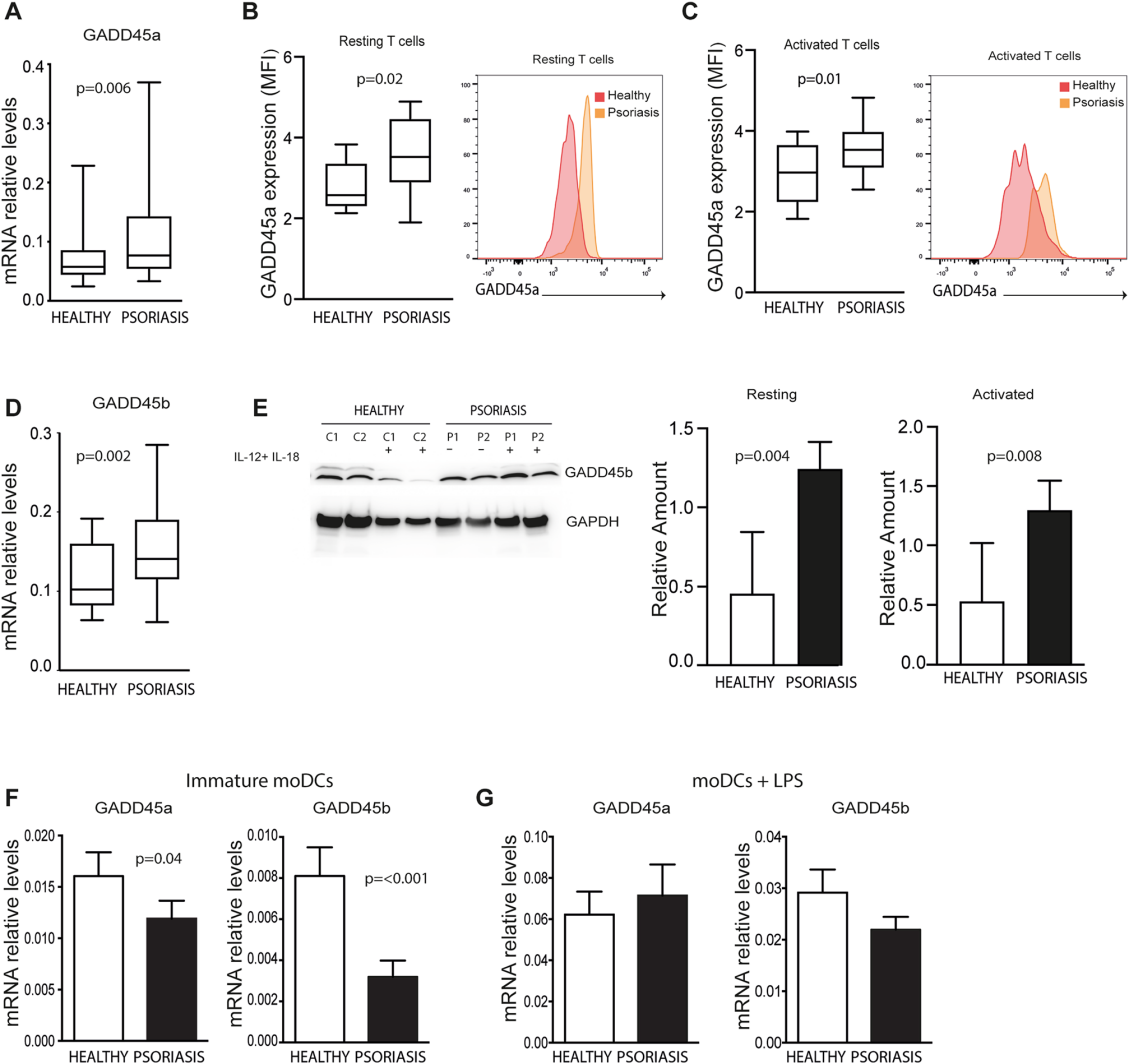
Differential expression of GADD45a and GADD45b in peripheral blood leucocytes from psoriasis patients and healthy subjects. (**A**) Box charts show mRNA expression of GADD45a in unstimulated CD4^+^ T cells from psoriasis patients (n = 20) and controls subjects (n = 15). Expression was analyzed using qRT-PCR and GAPDH was used to normalize data. (**B**) Box charts show the expression of GADD45a in resting T cells from psoriasis patients (n = 14) and controls (n = 9). (**C**) Box charts show the expression of GADD45a in activated T cells from psoriasis patients (n = 14) and controls (n = 9). PBLs were incubated in the presence of anti-CD3/CD28 for 16 h, then GADD45a expression was evaluated by flow cytometry on CD3^+^ T cells. **B-C**, Data correspond to mean fluorescence intensity (MFI) of GADD45a expressed on gated CD3^+^ T cells. Representative histograms are shown. (**D**) GADD45b expression in unstimulated CD4^+^ T cells as in A. (**E**) Expression of GADD45b in total lysates of monocyte-free PBLs from psoriasis patients. Cells were incubated 24 h in the presence or absence of a mixture of IL-12 and IL18 (10 ng/ml). Representative blots from 2 out 6 patients and 2 out of 6 controls, and densitometric quantifications are shown. Bar charts show relative levels of GADD45b (data normalized to GADPDH). (**F**) Bar charts show GADD45a and GADD45b mRNA expression levels in immature moDCs analyzed using qRT-PCR. GAPDH was used to normalize data. (**G**) Expression of GADD45a and GADD45b mRNA in moDCs after 24 h of stimulation with LPS (10 ng/ml). Differences between groups were determined using Mann–Whitney U test.

Next, we evaluated the expression of GADD45a and GADD45b in peripheral blood lymphocytes. RT-PCR assays showed that CD4^+^ T cells from psoriasis patients expressed higher levels of GADD45a compared to control subjects (Fig. 3A). Flow cytometry analysis confirmed the higher expression of the GADD45a protein in peripheral T lymphocytes from psoriasis in both, unstimulated T cells and TCR-activated T cells (Fig. 3B-C). Circulating CD4^+^ T lymphocytes from patients with psoriasis were also shown to have increased levels of GADD45b mRNA (Fig. 3D) and protein (Fig. 3E). Expression of GADD45a and GADD45b was also studied following stimulation with pro-inflammatory cytokines, showing and induction of GADD45b but not of GADD45a (Supplementary Fig. 3). T cells from psoriasis patients expressed higher levels of GADD45b after stimulation with a mixture of IL-12 and IL-18 compared to controls (Fig. 3E). Besides T cells, DCs play a key role during different phases of the development of psoriasis. Although the expression of GADD45a and GADD45b molecules was diminished in non-stimulated moDCs from psoriasis patients compared to controls (Fig. 3F), after LPS stimulation both patients and controls were able to upregulate the expression of GADD45a and GADD45b at similar levels (Fig. 3G).

**Figure 4:**
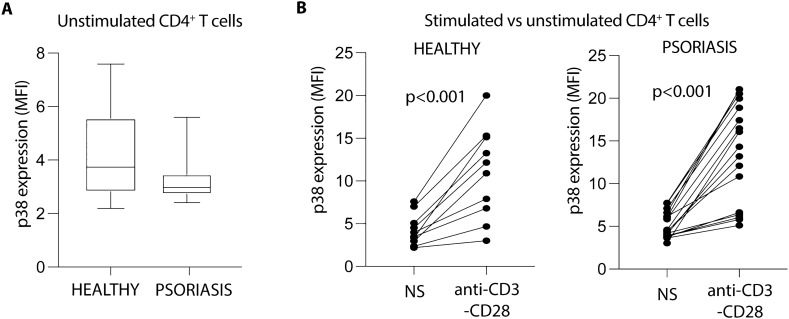
p38 activation is not affected in CD4^+^ T cells from psoriasis patients. (**A**) Dual phosphorylation of p38 was evaluated by flow cytometry in peripheral blood CD4^+^ T cells from psoriasis patients (n = 16) and healthy subjects (n = 10), immediately after isolation. Data were analyzed using Mann–Whitney U test. (**B**) Activation of p38 before and after TCR stimulation. T cells were either left untreated (not stimulated, NS) or activated in the presence of anti-CD3 (10ug/ml) and anti-CD28 (5ug/ml) antibodies during 15 min. Thereafter, dual phosphorylation of p38 was evaluated by flow cytometry as in A. Data were analyzed using Wilcoxon matched signed rank test.

Next, we wonder whether the differential expression of GADD45 proteins in T cells could be affecting the signaling pathway of p38 in psoriasis patients. Analysis of the activation of p38 in basal conditions and following TCR stimulation in patients and controls showed no statistical differences neither in resting (Fig. 4A) nor in anti-CD3/CD28 stimulated cells (Fig. 4B). These data indicate that the increased expression of GADD45a and GADD45b does not influence the p38 activation pathways in CD4^+^ T cells from psoriasis patients.

### The UCHL1 promoter is hypermethylated in psoriatic lesional skin correlating with lower levels of mRNA transcript

**Figure 5:**
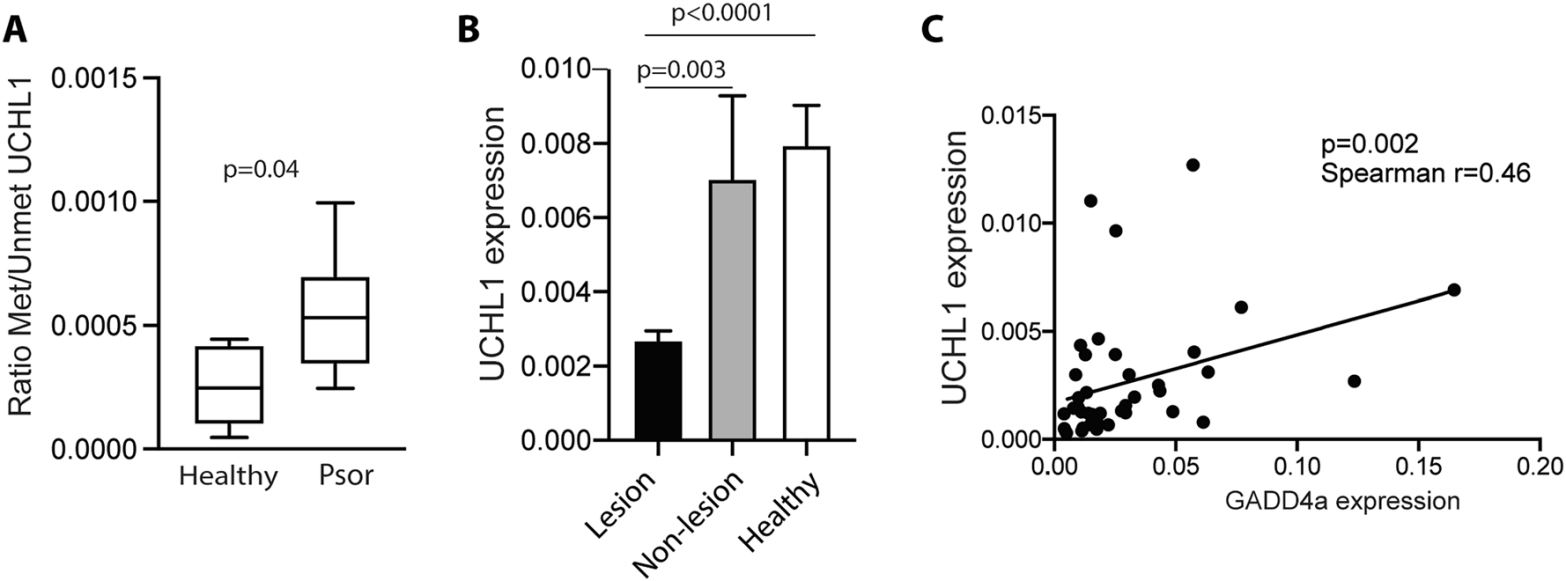
The promoter region of UCHL1 in lesional skin of psoriasis patients show a higher index of methylation. (**A**) Methylation ratio of the promoter region of UCHL1 in lesional skin samples from psoriasis patients (n = 6) and healthy skin from control subjects (n = 5). Methylated status of UCHL1 promoter was analyzed using methylation specific PCR. Data were analyzed using Mann Whitney U test. (**B**) Expression of UCHL1 in skin samples from psoriasis patients (lesional and non-lesional) and control subjects. UCHL1 expression was analyzed using qRT-PCR and data were normalized to GADPH. Data were analyzed using One-way ANOVA and Tukey’s multiple comparisons test. (**C**) Scatter graphs showing the positive correlation between the levels of GADD45a and UCHL1 in lesional skin of psoriasis patients. Data were analyzed using Spearman test.

It is known that GADD45a has a demethylase activity^25^ and its depletion results in a hypermethylation of the promoters of several genes such as UCHL1^14^. To determine the UCHL1 methylation state we used publicly available datasets, containing methylation microarray information from psoriasis skin samples. Among analyses of the differential methylation sites (DMS) between psoriasis skin and controls, we have focused on those significant DMS located in CpG islands of the UCHL1 promoter (Table 2). As expected, most of these sites are hypermethylated in psoriasis skin samples with respect to controls. These data were confirmed by methylation-specific PCR in lesional skin samples from psoriasis patients and healthy controls. Our data showed a higher methylation ratio of the promoter region of UCHL1 in psoriatic skin (Fig. 5A). Expression of UCHL-1 was significantly lower in lesional skin compared to both non-lesional skin of psoriasis patients and skin of controls (Fig. 5B). Moreover, a positive correlation between the levels of GADD45a expression and its target UCHL-1 was observed (Fig. 5C).

**Table 2:** Differentially methylated CpG sites in UCHL1 promotor in skin samples from psoriasis patients. Location: genomic coordinate_37 of the CpG site interrogated by the probe. CpG-site neighborhood: location of the gene-associated CpG-site(s) within the CpG-site neighborhood.

Dataset	Reference	DMS	Location	CpG-site neighborhood	Adj *p* value	Gene context	log2 FC	Methylation state
GSE63315 (L vs HC)	1	cg16026922	**41,259,044**	**Island**	1,38E-03	Body	5,68E−02	Hypermethylated
		cg16142306	**41,258,935**	**Island**	1,94E-03	1stExon;5'UTR	3,70E−02	Hypermethylated
GSE73894 L vs HC	2	cg24715245	**41,258,794**	**Island**	1,49E-03	TSS200	− 3,22E−02	Hypomethylated
		cg09921610	**41,259,866**	**Island**	6,56E-03	Body	2,95E−02	Hypermethylated
GSE73894 L vs NL		cg09921610	**41,259,866**	**Island**	6,06E-03	Body	− 3,50E−02	Hypomethylated
GSE115797	3	cg07068756	**41,258,910**	**Island**	1,05E-02	1stExon;5'UTR	7,52E−02	Hypermethylated

Gene context: location of the gene-associated CpG-site(s) with respect to the gene context. Abbreviations: Adj *p* value: adjusted p value; CpG: Cytosine-Phosphate-Guanine sites; DMS: differentially methylated sites; HC: Healthy control skin; Log2FC: logarithm of the fold change of methylation values in psoriasis samples with respect to controls; L: psoriasis lesional skin; NL: psoriasis non-lesional skin; TSS200 Promoter regions 200 bp (base pairs) upstream of the transcription start site; 5’-UTR: 5’-untranslated region; vs: versus.

## Discussion

Members of the GADD45 family of proteins are involved in numerous biological processes, many of which seem to be closely related to the pathogenesis of psoriasis. The data provided in this work show a complex dysregulation in the patterns of expression of these molecules among cellular players with essential functions in psoriasis, such as keratinocytes, DCs and T lymphocytes. While epidermal cells from these patients express low levels of GADD45a, infiltrating lymphocytes and peripheral CD4^+^ T cells exhibit a higher expression of these molecules compared to controls. A similar pattern seems to occur for GADD45b, revealing the complexity of GADD45 molecules, where different stimuli have been associated with cell-specific regulation of their expression. We detected a positive correlation between the expression of GADD45 molecules and the levels of IFN-γ. The pro-inflammatory environment in psoriatic skin could account for the increased expression of GADD45a and GADD45b. However, this statement seems to apply to leukocytes but not to keratinocytes.

The study of GADD45 proteins in human autoimmune diseases is barely explored. Polymorphisms in GADD45a and GADD45b genes and protein expression have been investigated in rheumatoid arthritis (RA) and systemic lupus erythematosus (SLE) patients. GADD45b but not GADD45a mRNA levels were significantly lower in RA patients compared to control cases. Regarding SLE, no differences in the expression of either molecule were detected^26^. Similar to the findings of RA patients, our data show that lesional skin from psoriasis patients express low levels of GADD45a and GADD45b. However, surprisingly a higher expression of IFN-γ was found in psoriasis patients. This unexpected inverse correlation has also been found in synovial fibroblasts of RA patients where a diminished expression of GADD45b was detected despite the higher activation of NF-kβ^27^. The absence of GADD45b has been associated with a higher expression of pro-inflammatory molecules in myeloid cells^3^, but the opposite has been described for GADD45a^6^. These findings support a complex counter-regulation of these molecules, where the outcome of the inflammation results from the balance between positive and negative signals. In psoriasis, the function of GADD45b and other anti-inflammatory signals seems to be overcomed by pro-inflammatory triggers. Unfortunately, we were unable to evaluate the expression of GADD45b by immunofluorescence, due to the lack of suitable detection reagents, and therefore cannot rule out a differential expression of this molecule in the different cells of the skin.

T cells are key players for the psoriasis development. Our data demonstrate that GADD45a and GADD45b are upregulated in peripheral CD4^+^ T cells from psoriasis patients at basal conditions. GADD45a and GADD45b show differences and similarities regarding the activation of T cells; while GADD45a acts as a negative regulator after TCR activation^5^, GADD45b seems to play a dual role favoring the activation of T cells at early time points but self-limiting cell activation during the chronic phase of inflammation^28^. Although the increment of both GADD45a/b molecules at basal conditions in psoriasis may be a consequence of the pro-inflammatory environment, we did not observe any difference in the activation of the p38 pathway compared to controls. The opposing effects of GADD45a and GADD45b molecules on T cell activation may counterbalance and account for the observed phenotype; the inhibitory effect of GADD45a on p38 activation seems to be overcome by the upregulated expression of GADD45b in psoriasis patients not only in unstimulated T cells but also following activation with IL-12 plus IL-18 cytokines. Further studies will be necessary to elucidate whether the increased expression of these molecules could be affecting the survival of activated CD4^+^ T cells.

GADD45 proteins act as stress sensors and are rapidly induced by genotoxic agents such as UV radiation and oxidative stress. In keratinocytes, GADD45a promotes cell cycle arrest, favoring genomic DNA repair and inhibiting cell death^11^. Recently, it has been reported that GADD45a silencing promotes cell proliferation and inhibits apoptosis in skin squamous cell carcinoma. Moreover, the silencing of GADD45a induced a local increased expression of cytokines such as IL-1, IL-6, TNF-α and VEGF^29^. It is conceivable that the reduction of GADD45a expression in keratinocytes from psoriasis patients could promote keratinocyte hyper-proliferation and the production of pro-inflammatory mediators.

Besides the above described functions, GADD45 proteins are involved in DNA demethylation. GADD45a interacts with components of DNA repair complexes, promoting their recruitment to specific sites and resulting in the replacement of methylated by unmethylated cytosines^25,30,31^. GADD45a depletion results in hypermethylation of UCHL1 promoter^14^. Accordingly, we have observed that most of the analyzed CpG sites located on UCHL1 promoter were hypermethylated in psoriasis skin samples with respect to controls. UCHL1, also known as PGP 9.5, is an enzyme with ligase and hydrolase activities, mainly expressed in neuroendocrine cells and the central nervous system. Our data demonstrate not only that the skin of psoriasis patients expresses lower levels of UCHL1 compared to controls, in agreement with previously published data^32^, but also a clear positive correlation with GADD45a expression. UCHL1 determines cellular levels of ubiquitins and glutathione and regulates cell cycle^33^. Interestingly, it is known that, in keratinocytes, UCHL1 inhibits the secretion of IL-8, IFN-I and MIP3, and suppresses the NF-kβ activity induced by TNF-α^34^. Its expression has also been associated to the suppression of iNOS induced by TNF-α and NFκ-β activation^35,36^. Here we propose that the reduction of GADD45a in the epidermis of psoriatic lesional skin is involved in the hypermethylation of UCHL1 promoter, then reducing the expression of this protein. This reduction in the levels of UCHL1 is related with an increase of the pro-inflammatory molecules, repressed under physiological conditions. This process may contribute to the inflammation in psoriatic skin. A better understanding of the mechanisms underlying psoriasis disease may provide new strategies for therapy and/or patient monitoring.

Psoriasis is a very complex disease with numerous deregulated molecules that participate in triggering a sustained activation of the immune system and epidermal cells, leading to the characteristic clinical manifestations of this disease. GADD45 proteins have been implicated in numerous biological processes and the finding that they relieve epigenetic gene silencing may account for some of the pleiotropic effects observed in the pathogenesis of psoriasis.

## Supplementary Information

Supplementary Information 1.

Supplementary Information 2.
