# Supplementary figures and images for: Growth arrest and DNA damage-inducible proteins (GADD45) in psoriasis
Source: Sci Rep. 2021 Jul 16;11:14579. doi: 10.1038/s41598-021-93780-x (PMC8285512; doi:10.1038/s41598-021-93780-x)

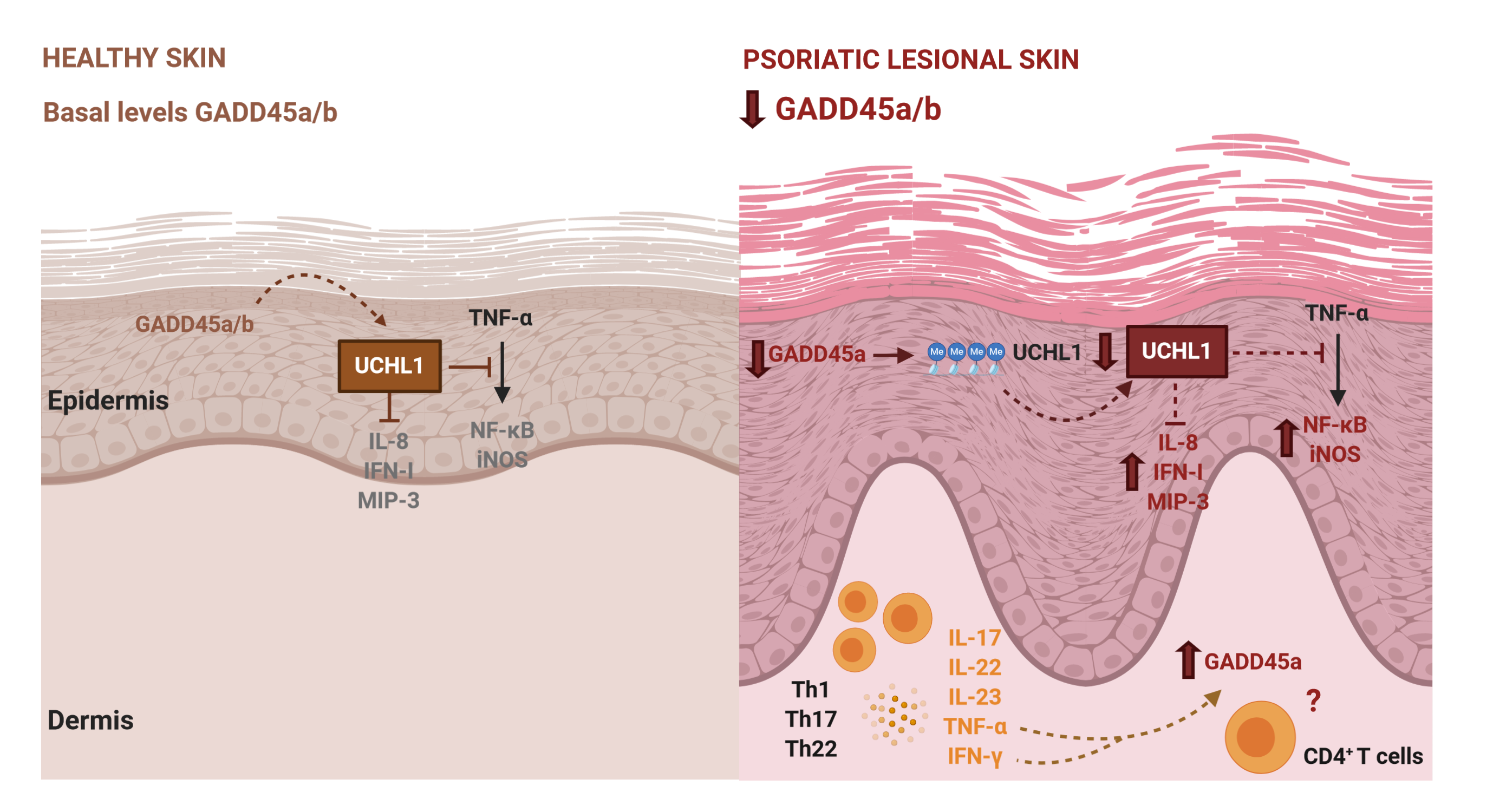

Supplement: Supplementary file 1 — Supplementary Information 1. [file 41598_2021_93780_MOESM1_ESM.png]
