# Supplementary material for: Growth arrest and DNA damage-inducible proteins (GADD45) in psoriasis
Source: Sci Rep. 2021 Jul 16;11:14579. doi: 10.1038/s41598-021-93780-x (PMC8285512; doi:10.1038/s41598-021-93780-x)
Supplement: Supplementary file 2 — Supplementary Information 2. [file 41598_2021_93780_MOESM2_ESM.pdf]

Supplementary Information

Growth Arrest and DNA Damage-inducible proteins (GADD45) in Psoriasis

Authors: Pedro Rodríguez-Jiménez[a,*], Lola Fernández-Messina[b,c,*], María C Ovejero-Benito[d,e,f], Pablo Chicharro[a], Paula Vera-Tomé[b], Alicia Vara[b], Danay Cibrian[g], Pedro Martínez-Fleta[b], María Jiménez-Fernández[b], Inés Sánchez-García[b], Mar Llamas-Velasco[a], Francisco Abad-Santos[d,e,f], Francisco Sánchez-Madrid[b,c], Esteban Dauden[a,#] and Hortensia de la Fuente[b,c,#].

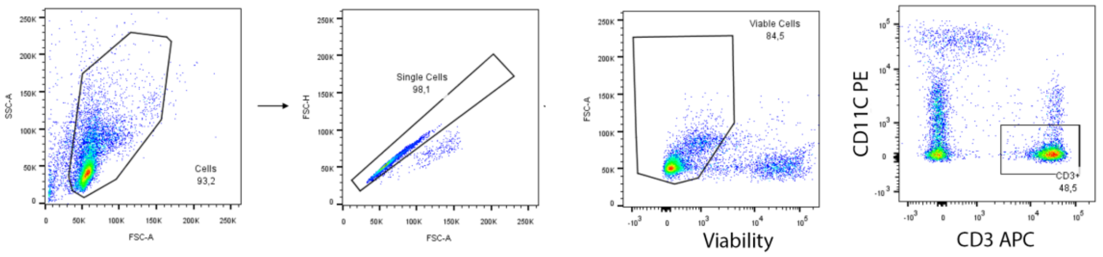

Supplementary Figure 1. Flow cytometry gating strategy. Expression of GADD45a was evaluated as mean fluorescence intensity on single, live, CD11c-CD3+ cells, as shown in the dot plots.

A.

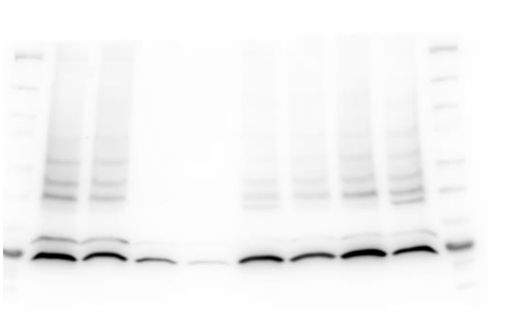

B.

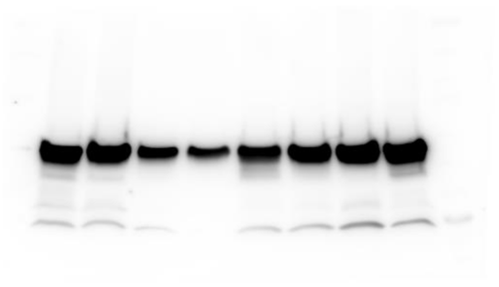

Supplementary Figure 2. Full length Western blots. *Expression of GADD45b* **(A)** *and GAPDH* ***(B)*** *in total lysates of monocyte-free PBLs from psoriasis patients. Cells were incubated 24 hr in the presence or absence of a mixture of IL-12 and IL18 (10ng/ml).*

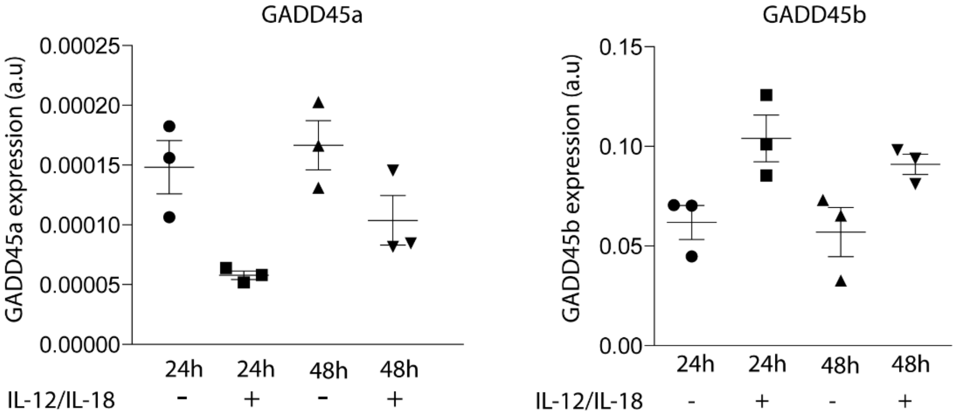

Supplementary Figure 3. Kinetic of GADD45a and GADD45b expression in T lymphocytes. CD4+ T cells were isolated from peripheral blood of healthy subjects and cultured in the presence or absence of a cocktail of IL-12 + IL-18 cytokines for the indicated times. Expression of GADD45a and GADD45b was evaluated by RT-PCR and normalized to the endogenous expression of GAPDH. Data correspond to mean±sd of three independent experiments.

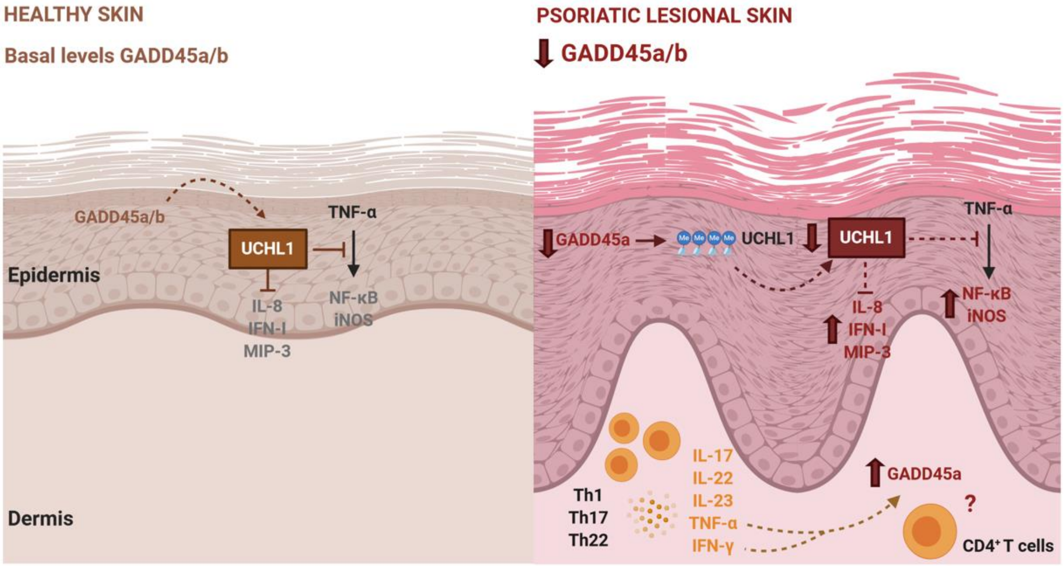

Graphical Abstract. A complex regulation of GADD45 proteins in psoriasis. Under non-pathological conditions, basal levels of GADD45a/b in the epidermis allow sustained levels of UCHL1 that suppress pro-inflammatory cytokines such as IL-8, and TNF-α dependent induction of NF-κB and iNOS. Here we propose that the reduction of GADD45a in the epidermis of psoriatic lesional skin is involved in the hypermethylation of UCHL1 promoter, then reducing the expression of this protein. This reduction in the levels of UCHL1 is related with an increase of the pro-inflammatory molecules, repressed under physiological conditions. This process may contribute to the inflammation in psoriatic skin. Furthermore, psoriasis is characterized by an infiltration of effector T cells in the dermis (Th1, Th17, Th22) that release pro-inflammatory cytokines, including TNF-α and IFN-ɣ, that could induce the upregulation of GADD45a in the inflammatory infiltrate. Whether the upregulation of GADD45a in T cells from psoriasis patients is involved in the inflammatory process characteristic of this disease, remains unknown. Altogether, GADD45 proteins participate in the inflammatory response in psoriasis, although further studies will be needed to fully understand the mechanisms involved in this complex regulation.
